# High performance of carbon nanotubes/silver nanowires-PET hybrid flexible transparent conductive films via facile pressing-transfer technique

**DOI:** 10.1186/1556-276X-9-588

**Published:** 2014-10-28

**Authors:** Mao-xiang Jing, Chong Han, Min Li, Xiang-qian Shen

**Affiliations:** 1 Institute for Advanced Materials, Jiangsu University, Xuefu road 301 Zhenjiang 212013, China

**Keywords:** Flexible transparent conductive film, CNTs/AgNWs, Adhesion, Pressing-transfer

## Abstract

To obtain low sheet resistance, high optical transmittance, small open spaces in conductive networks, and enhanced adhesion of flexible transparent conductive films, a carbon nanotube (CNT)/silver nanowire (AgNW)-PET hybrid film was fabricated by mechanical pressing-transfer process at room temperature. The morphology and structure were characterized by scanning electron microscope (SEM) and atomic force microscope (AFM), the optical transmittance and sheet resistance were tested by ultraviolet-visible spectroscopy (UV-vis) spectrophotometer and four-point probe technique, and the adhesion was also measured by 3M sticky tape. The results indicate that in this hybrid nanostructure, AgNWs form the main conductive networks and CNTs as assistant conductive networks are filled in the open spaces of AgNWs networks. The sheet resistance of the hybrid films can reach approximately 20.9 to 53.9 Ω/□ with the optical transmittance of approximately 84% to 91%. The second mechanical pressing step can greatly reduce the surface roughness of the hybrid film and enhance the adhesion force between CNTs, AgNWs, and PET substrate. This process is hopeful for large-scale production of high-end flexible transparent conductive films.

## Background

Flexible transparent conductive films (FTCFs) have received much attention because of their electrical and optical properties and their feasibility in bending, folding, and mounting to a surface, which have a great potential to be applied in a large-area display, touch screen, light-emitting diode, solar cell, semiconductor sensor, etc. [1-7]. Indium tin oxide (ITO) as a traditional transparent conductive material has been widely used for organic solar cells and light-emitting diodes; however, it cannot meet the market demand of FTCF due to its rising cost and brittleness and hence it has limited applicability in flexible electronic devices [8-10]. Carbon nanotubes (CNTs) [11,12], graphene [13,14], or a hybrid of them [15] have attracted significant interest and have been successfully used as transparent conductive materials on flexible substrates in organic light-emitting diodes and solar cells. However, their performance in terms of sheet resistance and transparency is still inferior to ITO. Metal nanowires (MNWs) are a promising replacement of ITO, CNTs, or graphene because of their high dc conductivity and optical transmittance [16,17]. Gold nanowire (AuNW) [18], silver nanowire (AgNW) [19-23], copper nanowire (CuNW) [24-27], aluminium nanowire (AlNW) [28], and hybrid [29,30] films have been demonstrated to have optical transmittance comparable to an ITO film at the same sheet resistance. Especially MNWs on a plastic substrate can have better mechanical properties than ITO.

Nevertheless, researchers found that MNW films have electrically nonconductive open spaces (approximately 200 to 1,000 μm), and the open spaces become bigger for sparser networks [31,32], and some applications require continuously conductive or low nonconductive regions. The large openings in a MNW network could be problematic for some device applications when the charge diffusion path length is less than the hole size. One strategy to overcome the defect of MNW films is to fill components such as graphene [32-34], CNTs [35], conductive polymers [36-39], or metal oxides [40], but these reported methods may cause processing and cost problems. Increasing the density of MNWs may also reduce the open spaces and the sheet resistance, but the optical transmittance may also be greatly affected. Meanwhile, the price of MNWs, especially AuNWs and AgNWs, is still too high to be heavily used for decreasing manufacturing cost. Significant improvement is needed for new materials or processes which can bring cost-effective and reliable transparent conductive films.

In this work, we attempted to mix and use CNTs and AgNWs as conductive materials and transfer CNT/AgNW hybrids on flexible polyethylene terephthalate (PET) film and then form CNT/AgNW-PET films by a facile two-step mechanical pressing technique. In this design, AgNWs were the main conductive networks, and CNTs as the assistant conductive networks were filled in the open spaces of the AgNW networks; both of them had good connections, which made the CNT/AgNW-PET films possess low sheet resistance and high optical transmittance.

## Methods

**Figure 1:**
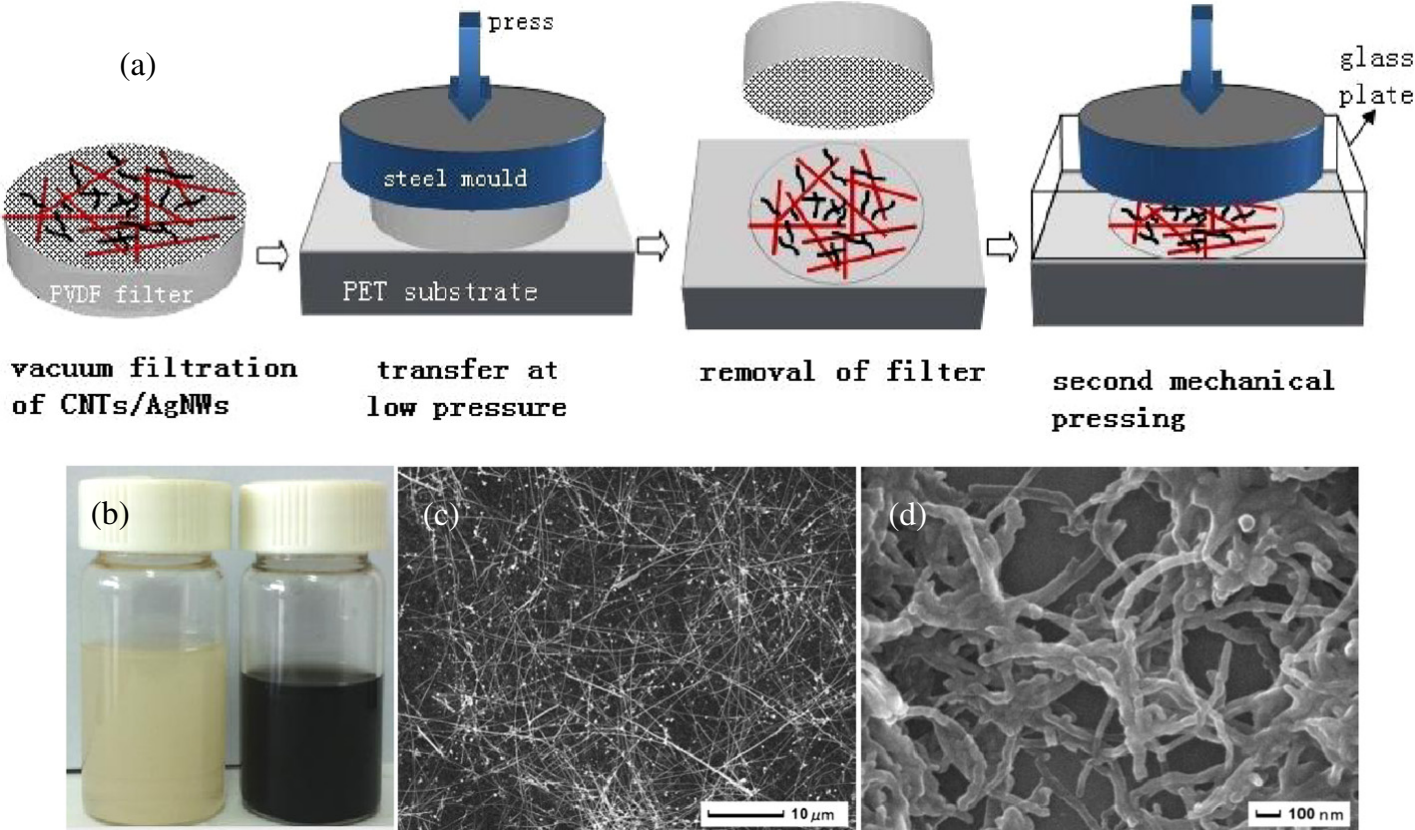
Transfer preparation of CNT/AgNW-PET films (a) and suspensions (b); SEM pictures of AgNWs (c) and CNTs (d).

The silver nanowires with a diameter of approximately 50 to 90 nm and a length of approximately 10 to 20 μm and the multi-walled carbon nanotubes with a diameter of approximately 20 to 50 nm and a length of approximately 5 to 15 μm used during the fabrication of the films were purchased from Nanjing Xianfeng Tech Co., Ltd (Nanjing, Jiangsu, China) and supplied with a concentration of 10 and 2 mg/mL in alcohol, respectively. The suspensions were further diluted to a concentration of 0.1 and 0.01 mg/mL, respectively, in alcohol which was subsequently used in all the transfer processes.The schematic representation of the preparation process of CNT/AgNW-PET films is shown in Figure 1a. First, the hybrid suspensions of CNTs/AgNWs with different ratios (mL/mL approximately 0.5/2, 1/2, 2/2, 4/2, 2/1, 2/3, 2/4) were obtained by direct mixing of CNT and AgNW suspensions, diluting to a volume of 10 mL and then supersonic dispersing treatment for 30 min. Then, the hybrid suspension was vacuum filtered by using a polyvinylidene fluoride (PVDF) filter membrane (Φ5 cm, hole diameter of 0.2 μm). Third, the hybrid CNT/AgNW film was transferred onto a PET substrate by pressing the filter membrane using a stainless steel plate and a press machine at a pressure of 3 MPa for 10 s. Then, the CNT/AgNW-PET film was obtained after lifting the pressure and removing the PVDF filter membrane slowly. When the semi-finished product was dried at room temperature for more than 30 min, to enhance the adhesion of CNT/AgNW networks on the PET substrate and reduce the junction resistance between CNTs and AgNWs, a second pressing at a pressure of 10 MPa for 30 s was implemented using a bare glass plate as a counter. As a comparison, a hybrid film was heated to 120°C for 30 min to test the effect of heating on the optical transmittance and sheet resistance.

Optical transmittance (*T*) was obtained using a Beijing PGeneral TU-1900 ultraviolet-visible spectroscopy (UV-vis) spectrophotometer (Beijing Purkinje General Instrument Co., Ltd., Beijing, China) with a blank PET as the reference. The surface morphology and structural pictures were obtained using a JEOL JSM-7001 field emission scanning electron microscope (SEM; JEOL Ltd., Tokyo, Japan) and Shanghai Zhuolun MicroNano D3000 atomic force microscope (AFM; Shanghai Zhuolun MicroNano Instrument Co., Ltd., China). Sheet resistance (*R*_s_) was measured using four-point probe technique by depositing silver paint with a thickness more than 80 nm at the corners in a square shape with sides of approximately 3 mm and at least ten locations across the sample, and the values reported in this work are the mean value obtained across the entire film. The adhesion test was carried out by observing the remaining nanowires adhering to the PET substrate and measuring the *R*_s_ and *T* of films when the 3M sticky tape was peeled off.

## Results and discussion

**Figure 2:**
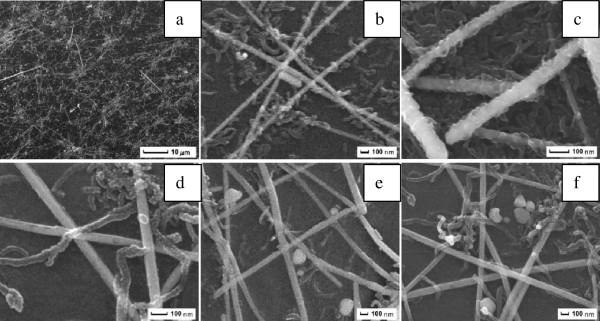
**SEM pictures of CNT/AgNW films at different ratios.** The different ratios are **(a)** and **(b)** 1:10, **(c)** 2:10, **(d)** 0.5:10, **(e)** 0.25:10, and **(f)** 1:15.

Figure 1a shows the schematic representation of the transfer process of CNT/AgNW networks onto the PET substrate. It can be found that this process has several distinguishing features. The entire process is implemented at room temperature and takes only several minutes. It is very critical for actual production due to avoiding the disadvantages from high temperature and complicated process. The process is also easy to control and adjust. First, the measured amount of CNTs and AgNWs are mixed in alcohol and sonicated for 30 min without adding any surface active agent that is enough to guarantee that the suspension is stable for more than 12 h, and the suspension and SEM pictures of AgNWs and CNTs are shown in Figure 1b,c. The nanowires can be dispersed very well by this method. Then, the suspension is filtered on a commercially available PVDF filter membrane to obtain a uniform film of CNTs/AgNWs. Whereafter, the PVDF membrane bearing the CNTs/AgNWs is pressed against the PET substrate at a moderate pressure of 3 MPa, because we found that in our experiments the sheet resistance of AgNW film has little change when pressed at a pressure of more than 3 MPa, so 3 MPa is enough for a AgNW film to reduce resistance. When the pressure is released after a few seconds and the PVDF membrane is peeled off slowly from the substrate, the CNT/AgNW film is entirely transferred onto the substrate. The size of the hybrid film is limited only by the size of the starting PVDF filter membrane. We note that the static pressing step can be replaced by rolling pressing to realize massive production. In the last step, a high pressure of 10 MPa is needed to enhance the junction between CNTs and/or AgNWs. Actually, the high pressure treatment is also very important to reduce surface roughness and adhesion of films that will be mentioned in the later section. In brief, these above-mentioned features are beneficial for the large-scale production of flexible transparent conductive films.Figure 2 shows the SEM pictures of CNT/AgNW films at different ratios on PET substrates fabricated with the mechanical pressing-transfer process. From Figure 2a, it can be seen that the transfer process is extremely uniform over the entire area of the film leading to a uniform density of nanowires everywhere on the substrate. With the different ratios of CNTs/AgNWs shown in Figure 2b,c,d,e,f, the AgNWs form the main conductive networks, and CNTs as the assistant conductive networks are filled in the open spaces of the AgNWs networks; both of them have good connections. The difference between them is the density of CNT networks due to the different addition amount of CNTs.

**Figure 3:**
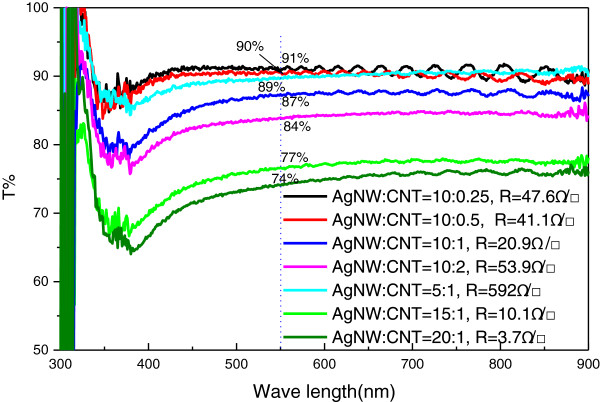
Optical transmittance and sheet resistance of CNT/AgNW-PET films by pressing-transfer process, a blank PET substrate was the reference.

The corresponding optical transmittance and sheet resistance of CNT/AgNW-PET films of several different ratios are shown in Figure 3. It can be seen that most of the films have a constant transmittance from 400 to 900 nm and low sheet resistance. When the adding amount of CNTs to AgNWs is approximately 0.25 to 2, the sheet resistance of hybrid films can reach approximately 20.9 to 53.9 Ω/□ with the optical transmittance of approximately 84 to 91% at *λ* = 550 nm (*T*_550_). Too much addition of CNTs or AgNWs would affect the sheet resistance and transmittance of hybrid films because of the absorption of visible light by CNTs and reflection by AgNWs [31]. Meanwhile, we note that when the amount of AgNWs is fixed and with the increase of CNTs from 0.5 to 2, the optical transmittance and sheet resistance of AgNW film have a relatively small change, while the amount of CNTs is fixed and with the increase of AgNWs from 5 to 20, the optical transmittance and sheet resistance of AgNW film have a distinct decrease simultaneously, so it can be concluded that the AgNW network plays a major part for the optical transmittance and sheet resistance of CNT/AgNW-PET films, while the CNT network just plays an assistant role.

**Figure 4:**
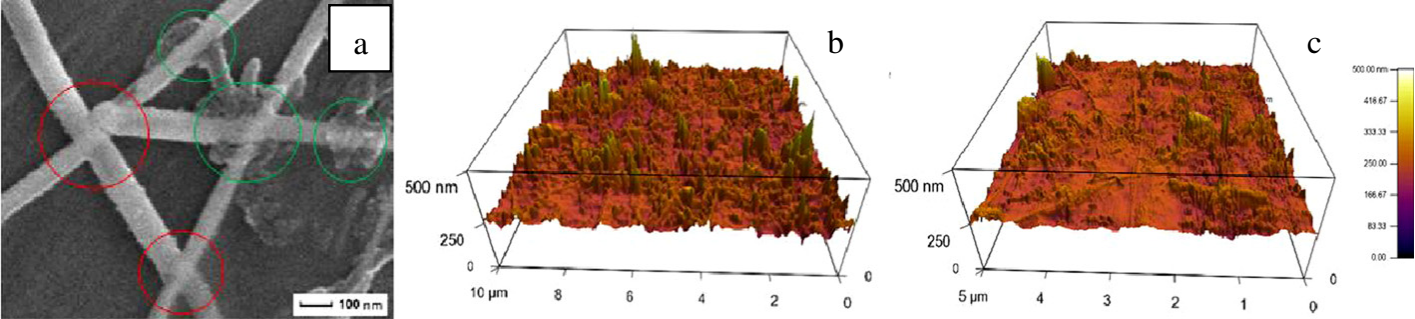
**SEM and AFM images of CNT/AgNW-PET film and CNT/AgNW network. (a)** SEM image of CNT/AgNW-PET film pressed at 10 MPa for 30 s; AFM images of the CNT/AgNW network **(b)** before and **(c)** after second pressing.

**Figure 5:**
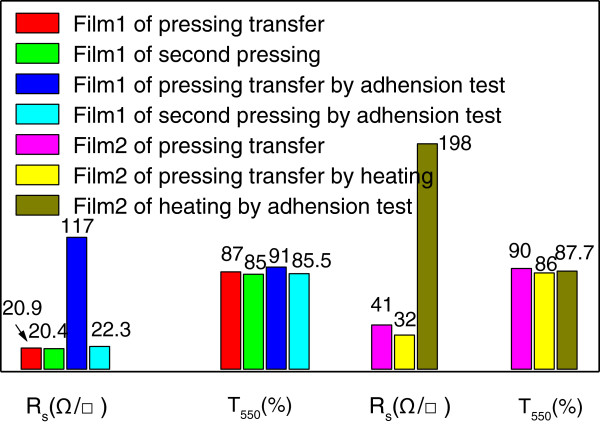
***R***_***s***_** and *T***_***550 ***_**of CNT/AgNW films before/after second mechanical pressing, adhesion test (3M sticky tape), and heating (120°C).**

For practical applications such as displays and solar cells, low roughness and enhanced adhesion are also always required [41-45]. In our study, these requirements were realized by second mechanical pressing at room temperature. Figure 4a shows the SEM image of a CNT/AgNW-PET film after pressing at 10 MPa for 30 s. The compressed, closely contact points between CNTs and/or AgNWs can be seen distinctly, and it will be beneficial for strong adhesion, low roughness, and junction resistance. As a consequence, the AFM images of the CNT/AgNW-PET film before and after second pressing in Figure 4b,c show that the surface roughness decreases greatly from 97.6 to 28.1 nm after second mechanical pressing. Adhesion tests were implemented by 3M sticky tape. Although it is enough for the hybrid film to reduce the junction resistance under 3 MPa, and second mechanical pressing has little effect on the transmittance and sheet resistance of the hybrid film as shown in Figure 5, we note that comparing with those without second pressing the hybrid film after second mechanical pressing has stronger adhesion to the PET substrate, and we tried to peel off the CNT/AgNW film from the PET substrate using 3M sticky tape by firmly attaching it on the surface of the CNT/AgNW film, but the film remained on the PET without visible change indicating its strong adhesion between CNTs/AgNWs and substrate. Meanwhile, from the results of *R*_s_ and *T*_550_ of the CNT/AgNW film before and after adhesion test, the *R*_s_ of the film without second pressing increases rapidly from 20.9 to 117 Ω/□; the *T*_550_ also changes from 87% to 91%. While with second pressing the *R*_s_ just increases from 20.4 to 22.3 Ω/□, the *T*_550_ changes from 85% to 85.5%. Therefore, we believe that the main role of the second mechanical pressing is reinforcing the adhesion force between CNTs, AgNWs, and PET substrate except for reducing surface roughness. To compare the effect of second pressing with traditional heating process [46], a film of pressing transfer without second pressing was heated at 120°C and also tested by 3M sticky tape as shown in film 2 of Figure 5. The *R*_s_ and *T*_550_ of film 2 by heating process decrease from 41 to 32 Ω/□ and 90% to 86%, respectively, while the *R*_s_ of the heated film after adhesion test rises dramatically to 198 Ω/□, which means that the heating process plays a role not as much as second mechanical processing for adhesion enhancement. Furthermore, comparing with other reported results, e.g., AgNW film with *R*_s_ approximately 20 Ω/□ and *T*_550_ approximately 80% prepared via meyer rod coating and pressing under 18 GPa by Cui's group [31], AgNWs film with *R*_s_ approximately 8.6 Ω/□ and *T*_550_ approximately 80% prepared via drop-coating and mechanical pressing under 25 MPa by Noji's group [45], and AgNW/CNT film with *R*_s_ approximately 17 Ω/□ and excellent stretchable property, but no *T* information prepared via vacuum filtration and plasmonic welding process by Woo and co-workers [35], our results and this simple technique have an obvious advantage and potential to be applied to practice from the economic and practical point of view.

## Conclusions

CNT/AgNW-PET flexible transparent conductive films were fabricated by mechanical pressing-transfer process at room temperature. AgNWs form the main conductive networks, and CNTs as the assistant conductive networks are filled in the open spaces of the AgNWs networks; both of them have good connections, and the sheet resistance of the hybrid films reaches approximately 20.9 to 53.9 Ω/□ with the optical transmittance of approximately 84 to 91%. The second mechanical pressing step can greatly reduce the surface roughness of the hybrid film and reinforce the adhesion force between CNTs, AgNWs, and PET substrate. This process is more hopeful to be used in practical production of flexible transparent conductive films compared with traditional heating-treatment process.

## Competing interests

The authors declare that they have no competing interests.

## Authors' contributions

SXQ designed the research, JMX performed the experiments and wrote the main manuscript text and prepared all figures, and LM and HC did some testing work and modified the manuscript and figures. All authors read and approved the final manuscript.
